# STAT6-dependent and -independent mechanisms in Th2 polarization

**DOI:** 10.1002/eji.201242433

**Published:** 2012-10-08

**Authors:** Elisabeth Maier, Albert Duschl, Jutta Horejs-Hoeck

**Affiliations:** Department of Molecular Biology, University of SalzburgSalzburg, Austria

**Keywords:** IL-2/STAT5 signaling, IL-4/STAT6 signaling, mTOR signaling, Notch signaling, Th2-cell polarization, Wnt signaling

## Abstract

Th2 cells play a key role in directing immune responses against helminths. Additionally, Th2 cells are crucial for many types of allergic reactions. Whereas the molecular mechanisms underlying the differentiation of other types of Th cells are well understood, Th2 differentiation is still a controversial topic. IL-4 and its downstream transcription factor signal transducer and activator of transcription (STAT)6 are well-known key mediators in Th2 differentiation. The fact that Th2 cells themselves are the most potent source of IL-4 suggests that additional mechanisms promoting the initiation of Th2 differentiation exist. This article gives an overview on STAT6-dependent and -independent mechanisms involved in the process of Th2 polarization, including Notch, mTORC2, IL-2/STAT5, and Wnt. Furthermore, we emphasize the role of STAT6 not only as a transcriptional activator promoting Th2 development, but also in fine-tuning alternative signaling pathways which are involved in the initiation of Th2 polarization.

## Introduction

The immune system is constantly confronted with self-antigens, innocuous antigens, and different kinds of foreign pathogens, and it must decide either to tolerate these or to elicit an immune response against them; furthermore, if a response is mounted, the immune system must decide what type of response is appropriate to clear the pathogen. To accomplish this task, naïve CD4+ T cells develop into regulatory T (Treg)-cell subsets, which protect the body from immune responses against auto-antigens, and into distinct subsets of effector cells, each of which is specialized to orchestrate an immune response against a different kind of pathogen. Based on the observation that CD4+ effector T cells help B cells to produce antibodies, they are also called T helper (Th) cells. The development of distinct CD4+ Th cell subsets out of an inexperienced mature cell pool requires three signals. The first signal is derived from antigen-presenting cells, usually dendritic cells, which present antigen-derived peptides in the context of MHC class II molecules to the T-cell receptor (TCR). The second, the so-called co-stimulatory signal, occurs following the interaction of the surface molecule B7 on dendritic cells with CD28 expressed on T cells. Polarizing cytokines constitute the third signal in CD4+ T-cell differentiation and determine the functional specification of the resulting CD4+ subsets. The signals derived from the polarizing cytokines lead to expression of specific transcription factors which, in turn, direct the expression of effector cytokines through which the CD4+ T cells exert their function and by which the distinct subsets can be identified. Despite the important role that CD4+ T-cell subsets play in host defense, they are also associated with severe immune pathologies, including autoimmunity, hypersensitivity, and tumorigenesis.

Th2 cells are specialized to elicit immune reactions against parasitic worms. They migrate to specific tissues where they recruit and activate a number of different cell types by releasing the cytokines IL-4, IL-5, IL-9 (now often assigned to the Th9 phenotype), IL-10, IL-13, IL-25, and IL-31 1–3. Yet, misguided Th2 responses result in the development of allergic disorders, which are a major health problem in industrialized countries 1. Whereas mechanisms controlling the differentiation of Th1 cells, Th17 cells, and regulatory T-cell subsets are well described, the issue of Th2 differentiation is still being debated. Thus, molecular mechanisms engendering the development of Th2-mediated inflammation are currently under intense investigation.

**Figure 1:**
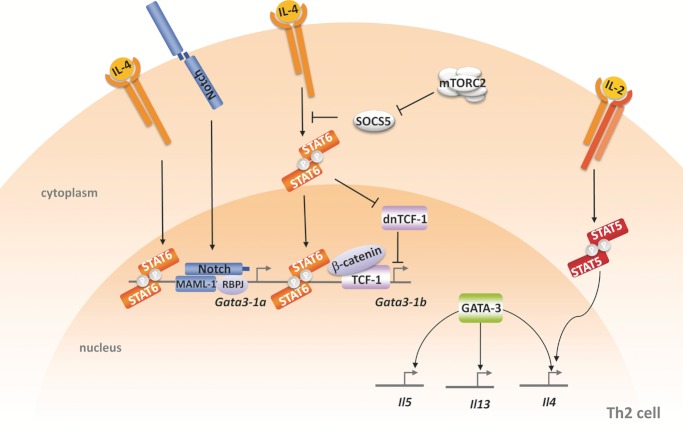
Signaling pathways in Th2 differentiation. Binding of IL-4 to the IL-4 receptor results in the phosphorylation and dimerization of signal transducer and activator of transcription 6 (STAT6). STAT6 dimers translocate into the nucleus where they promote expression of the Th2 master regulator GATA-binding protein 3 (GATA3). GATA3 is translated from two distinct transcripts termed GATA3–1a and GATA3–1b that derive from two different promoters. Activated STAT6 induces the expression of GATA3 from both promoters. GATA3, in turn, binds to and modifies the IL5–IL13–IL4 locus, which results in enhanced expression of Th2-related cytokines. Additionally, several other pathways facilitate Th2 differentiation. Suppressor of cytokine signaling 5 (SOCS5) acts as a repressor of STAT6-mediated transcription. The mTORC2-signaling complex supports IL-4/STAT6 signaling by inhibiting SOCS5. IL-2 contributes to Th2 differentiation by activating STAT5A, which induces transcription of IL-4 in a GATA3-independent way. Upon activation of Notch, the intracellular Notch-domain forms a signaling complex with RBPJ (recombination-signal-binding protein for immunoglobulin-κ J region) and Mastermind-like 1 (MAML-1), which induces transcription from Gata3–1a. T-cell factor 1 (TCF-1) and β-catenin induce GATA3–1b transcription in a STAT6-independent way. Activation of GATA3 by TCF-1 is counterregulated by dominant-negative isoforms of TCF-1 (dn TCF-1) which are also expressed in naïve T cells. Low concentrations of IL-4 inhibit the expression of the dominant-negative TCF-1 isoforms thereby promoting TCF-1-induced GATA3 expression and Th2 polarization.

For a long time, immunologists investigating the process of Th2 differentiation faced a “Th2 paradox”: On the one hand, IL-4 is the major Th2 cytokine and it was clear that Th2 cells require IL-4 and its downstream effector signal transducer and activator of transcription (STAT)6 for development 4. On the other hand, the only known source for IL-4 in those days was the Th2 cells themselves. Today, other cell types, namely basophils 5, 6, mast cells 7, 8, NK T cells 9, 10, follicular T helper cells 11 and naïve CD4+ cells 12, are thought to serve as sources for IL-4. Moreover, a number of recent studies provide evidence that pathways independent of IL-4/STAT6 signaling may induce Th2 differentiation in a complementary way 13–18. The present article gives an overview on IL-4/STAT6-dependent and -independent signaling pathways involved in Th2 differentiation (Fig. 1) and illustrates that the well-coordinated interplay of different signaling pathways is essential to obtain optimal Th2-cell development.

### IL-4/STAT6 signaling

The first-described and best-characterized pathway promoting Th2 fate is the IL-4-signaling cascade, involving the transcription factor STAT6 19, 20. IL-4 exerts its function by binding to its cognate receptor, the type-I IL-4R consisting of the IL-4 receptor alpha chain (IL-4Rα) and the common gamma chain (γc). Binding of IL-4 to IL-4Rα entails dimerization of the two receptor subunits and leads to phosphorylation of tyrosine residues within IL-4Rα by Janus Kinases. STAT6 monomers bind via their src homology 2 domains to the phospho-tyrosine residues within the intracellular portion of IL-4Rα and become phosphorylated by the juxtaposed Janus Kinases. Subsequently, phosphorylated STAT6 monomers dimerize via their src homology 2 domains and then translocate into the nucleus where they regulate the expression of IL-4 target genes. STAT proteins recognize the sequence motif TTC(N)_2–5_GAA 20. STAT6 preferentially binds to STAT6 motifs in which the palindromic half-sites TTC/GAA are divided by four arbitrary bases 21. Like other STAT proteins, STAT6 acts as a transcriptional activator for the vast majority of its target genes (hence the name “signal transducer and activator of transcription”). Although STAT6 mainly promotes gene expression, several studies suggest that STAT6 has inhibitory actions as well. The first reports on STAT6-mediated inhibition showed that the repressor function of STAT6 exerts its effect by occupying overlapping binding sites of other transcription factors, thereby sterically hindering their ability to bind and thus activate their target genes 22, 23. It is now acknowledged that STAT-mediated repression is a more common feature than previously thought and plays a role in the lineage commitment of Th subsets 24. STAT6 binds to the genomic loci of Th1-associated genes and promotes histone modi-fications related to more condensed chromatin, whereas STAT4 acts on Th2-associated loci in a similar way 25.

Once activated, STAT6 promotes expression of the Th2 master regulator GATA-binding protein 3 (GATA3), which is responsible for expression of Th2 effector cytokines. GATA3 is translated from two distinct transcripts, *GATA3–1a* and *GATA3–1b*, derived from two different promoters. Upon TCR stimulation in the absence of IL-4, *GATA3–1a* transcripts are not expressed and *GATA3–1b* expression is drastically reduced 26. In Th2 differentiation, both transcripts from the *GATA3* locus are expressed, albeit at different stages of Th2 development. *GATA3–1b* transcripts are detected early after Th2 induction (within hours), whereas transcripts derived from the proximal promoter occur with a delay of 2–4 days 27. IL-4/STAT6 signaling is critically involved in the activity of both *GATA3* promoters and thus controls both the onset and maintenance of *GATA3* expression. STAT6 is not only implicated in the initiation of Th2 differentiation, but it also contributes to maintenance of the Th2 phenotype by inducing the expression of chemokines and other Th2-relevant genes 20, 28–31.

A series of knockout studies clearly demonstrates the role of IL-4 and STAT6 in the development of Th2 cells. In mice defective for IL-4, Kopf et al. observed impaired Th2 responses, characterized by reduced Th2 effector cytokine production, loss of IgE class switching, and reduced eosinophilia upon infection with *Nippostrongylus brasiliensis* 32. Similar observations, though with a more pronounced phenotype, were reported in four independent studies involving STAT6−/− mice [4, 33–35]. The role of IL-4/STAT6 signaling in Th-2-mediated allergic inflammation has been extensively reviewed by Kuperman and Schleimer 36.

Two recent studies impressively demonstrated the importance of STAT6 in human and murine Th2-cell development. Elo et al. identified 453 genes that are regulated by STAT6. Remarkably, they found the expression of more than 80% of IL-4-mediated genes to be controlled by STAT6 24. Wei et al. analyzed STAT6-bound genes in developing mouse Th2 cells. They showed that STAT6-binding sites often co-localize with histone modifications that mark accessible chromatin and that some of these modifications are clearly dependent on the presence of STAT6 37. Thus, STAT6 does not only act as a transcription factor at the promoters of target genes, but also orchestrates epigenetic marks that favor the expression of Th2-relevant genes.

STAT6 is not the only STAT protein involved in Th2 polarization. STAT5A (see *IL-2/STAT5 pathway*) 38 and STAT3 39 were also reported to contribute to the Th2 phenotype. However, STAT3 and STAT5 activate genes that play a role in other CD4+ T-cell subsets as well and therefore they are not exclusively involved in Th2 differentiation.

STAT6 also contributes to the development of another effector CD4+ T-cell subset, namely Th9. As in Th2 cells, Th9-cell differentiation depends on IL-4, STAT6, and GATA-3, but besides IL-4 TGF-β1 is also required for polarization. Th9 cells are characterized by high expression of the transcription factor PU.1 40 and secretion of the cytokine IL-9 (hence the name) and in mice also IL-10, but they do not produce the typical Th2 cytokines IL-4, IL-5, or IL-13. However, similar to Th2 cells, Th9 cells are implicated in immune responses against parasites and in allergy 41.

### Notch-signaling pathway

Although STAT6 signaling is widely accepted to be crucial in Th2 polarization, studies demonstrating that STAT6−/− mice still display Th2 responses 17, 42 suggest that STAT6-independent mechanisms involved in Th2 polarization also exist. One pathway directing various differentiation programs, including T-cell differentiation, is the Notch-signaling pathway. Notch is a hetero-dimeric, membrane-bound receptor consisting of an extracellular domain and a single-pass transmembrane domain. There are four mammalian Notch proteins, termed Notch 1 to Notch 4, all of which are expressed by the CD4+ T-cell compartment. Two protein families that serve as ligands for Notch, the Jagged family, consisting of Jagged 1 and Jagged 2, and the delta-like ligands (DLLs), composed of DLL1, DLL3, and DLL4, have been described 43. Delta–Notch interactions (DLL1 or DLL4 and Notch 3) promote the Th1 phenotype, whereas Jagged-binding (Jagged 1 or Jagged 2 with Notch 1 or Notch 2) initiates Th2-cell development. Upon ligand binding, the Notch receptor is cleaved in its transmembrane region, causing the release of its intracellular domain from the plasma membrane. The intracellular domain translocates to the nucleus where it activates transcription of Notch target genes by associating with the DNA-binding factor RBPJ (recombination-signal-binding protein for immunoglobulin-κ J region), co-activators such as Mastermind-like 1 and chromatin-modifying enzymes 43. The importance of Notch signaling for Th2 cells became evident when different knockout mice were analyzed. Mice defective in either RBPJ or Notch 1 and Notch 2 showed impaired Th2 responses to parasite antigens 15. This is due to the fact that Notch signals direct the expression of *IL4* and *GATA3*. Several RBPJ-binding sites were identified within the 3′ enhancer of the *IL4* gene and the upstream promoter of *GATA3* (termed *GATA3–1a*) 14, 15. Noteworthy, these binding sites are juxtaposed to GATA3-binding sites. As GATA3 and Notch were shown to synergize in order to promote Th2 differentiation, it is likely that GATA3 and Notch are recruited to the *GATA3* and *IL4* loci in a cooperative fashion by associating with each other. It is clear that Notch signaling substantially contributes to Th2-cell development; however, defects in Notch signaling can, at least in vitro, be compensated by treatment with excess IL-4 to yield normal Th2 cells 43. The Notch ligand DLL4, a key molecule in Th-cell differentiation was reported to modulate disease pathogenesis during allergen-dependent inflammation and respiratory viral infection. Jang and colleagues observed enhanced allergic lung inflammation and an increase in Th2 cytokine production upon DLL4 blockade 44. A second study demonstrated that neutralization of DLL4 during respiratory syncytial viral infection increased Th2 cytokine production as well 45. Both studies indicate that DLL4 may support a Th1 environment by inhibiting Th2 cytokine responses.

### mTORC2

mTOR (mammalian target of rapamycin) is a member of the phosphatidylinositol-3OH-kinase (PI(3)K)-related kinase family and is involved in the regulation of metabolism, CD8+ T-cell memory and lymphocyte trafficking 46. Recently, Delgoffe et al. reported that mTOR contributes to Th-cell development by forming two distinct signaling complexes that play discrete roles in Th-cell polarization. The first mTOR-signaling complex (mTORC1) — assembled by mTOR, the scaffolding protein Raptor (regulatory-associated protein of mTOR), mammalian lethal with Sec13 protein 8 (mLST8), proline-rich AKT substrate 40 kDa (PRAS40) and dishevelled, egl-10, pleckstein (DEP)-domain-containing mTOR-interacting protein (Deptor) — mediates Th1 and Th17 differentiation. The second mTOR complex (mTORC2) contains, besides mTOR and mLST8, the scaffolding protein Rictor (rapamycin-insensitive companion of mTOR), mammalian stress-activated protein kinase-interacting protein (mSIN1), and protein observed with Rictor-1 (Protor-1), and promotes Th2 development 13, 47. The mTORC2 complex contributes to Th2 differentiation by down-regulating the negative feedback inhibitor suppressor of cytokine signaling 5 13. As suppressor of cytokine signaling 5 was shown to suppress STAT6 activation 48, down-regulation of this inhibitor contributes to an increase in STAT6 activity and consequently to enhanced Th2 differentiation. Upon disruption of mTORC2 T cells failed to differentiate into Th2 cells, characterized by the inability to up-regulate *GATA3* and IL-4 expression, whereas Th1 differentiation was barely affected. On the contrary, inhibition of mTORC1 resulted in a Th2-skewed phenotype. Interestingly, inhibition of both signaling complexes induces a Treg phenotype 13. A similar study conducted by Lee et al. gave in some aspects different results. Although disruption of mTORC2 also leads to a marked inhibition of Th2 development in this report, Th1 differentiation was affected as well, although to a minor extent. When the authors tried to restore Th1 and Th2 phenotypes in these cells they observed that constitutive active Akt rescued Th1 development, whereas constitutive PKC-θ was required to retrieve Th2 development. This indicates that the decision if a cell enters the Th1- or the Th2-differentiation program is made by different signals downstream of mTORC2 49.

### IL-2/STAT5 pathway

IL-2 is a cytokine that stimulates the proliferation of CD4+ T cells. In association with TGF-β1, IL-2 induces a regulatory phenotype. However, IL-2 and IL-2-induced STAT5A activation were also shown to induce the expression of IL-4 and hence to provide a mechanism of Th2 priming 38, 50. Constitutive activation of STAT5A was reported to elicit the production of IL-4 even in the absence of IL-4Rα and STAT6 50.

Usually, Th2 differentiation is accompanied by up-regulation of GATA3. Yet, modification of STAT5A activity does not influence GATA3 expression. When constitutively active STAT5A is introduced to naïve T cells, they produce IL-4 even if they are cultured under conditions that favor Th1 development 50. Blocking of IL-2 in Th2 cultures markedly impairs IL-4 production by these cells, but does not affect GATA3 levels. Furthermore, over-expression of GATA3 failed to compensate defects in STAT5A in an attempt to retain IL-4 production 38. Thus, IL-2/STAT5A and GATA3 are independent of each other. Nonetheless, they synergistically regulate IL-4 production. In vivo studies reinforce the role of STAT5A in Th2-mediated inflammation. Compared to STAT6−/− mice, STAT5A−/−STAT6−/− mice showed a significant reduction in Th2 development and a clear decrease in Ag-induced recruitment of eosinophils and leucocytes into the airways 51. This suggests that STAT5A plays an indispensable role in STAT6-independent Th2 development and Th2-mediated inflammation. More recently it was demonstrated that for thorough Th-cell differentiation, in addition to activation of a specific STAT by polarizing cytokines, a further cytokine signal provided by an IL-1 family member is required 52, and that IL-33 supports the IL-2/STAT5A-signaling pathway in generating high amounts of Th2 cytokines. Moreover, the IL-33 receptor was shown to be a target of IL-2. Thus, IL-2 makes T cells susceptible to IL-33 treatment and IL-2 and IL-33 then synergistically up-regulate the expression of Th2 cytokines.

### Wnt-signaling pathway

The canonical Wnt-signaling pathway involves the transcription factors T-cell factor 1 (TCF-1) and lymphoid enhancer-binding factor 1 (LEF-1), which reside in the nucleus where they are bound to Wnt-response elements. To activate target gene expression, TCF-1 and LEF-1 must first associate with β-catenin. In the absence of Wnt ligand, free β-catenin is constantly degraded by an adenomatous polyposis coli/Axin/glycogen synthase kinase 3 β (GSK3β)-protein complex. Upon binding of a Wnt ligand to the receptor, the β-catenin degrading complex dissociates, and free β-catenin accumulates and translocates into the nucleus where it displaces transcriptional repressors attached to the Wnt pathway transcription factors. As a consequence, β-catenin recruits the transcriptional machinery and the Wnt target genes are expressed 53. Wnt signaling plays an important role in many developmental processes, ranging from embryonic development to T-cell development in the thymus. More recent data provide evidence that Wnt signaling is also involved in Th2 polarization. For example, LEF-1 blocks binding of GATA3 to the *IL5* promoter 54 and acts as a repressor of *IL4* transcription 55. Therefore, LEF-1 is considered a negative regulator in Th2-associated immune responses. Similar to LEF-1 and TCF-1, the special AT-rich-binding protein 1 is able to activate gene transcription in association with β-catenin. Special AT-rich-binding protein 1 was shown to contribute to Th2 lineage commitment via inducing GATA3 expression in a Wnt-/β-catenin-dependent way 56.

Recently, TCF-1 was ascribed a key role in Th2-cell development. Yu et al. demonstrated that upon TCR activation, TCF-1 and β-catenin stimulate the expression of *GATA3–1b* and, as a consequence, IL-4 production 16. The fact that TCF-1 induced *GATA3–1b* transcription in CD4+ T cells derived from STAT6−/− mice to a level similar to that in wild-type control cells indicates that TCF-1 contributes to Th2 polarization in a STAT6-independent way. Besides promoting Th2 polarizing mechanisms through induction of GATA3 expression and subsequent release of IL-4, TCF-1 also acts as a repressor of the Th17 cytokine IL-17 57 and inhibits the production of the Th1 cytokine interferon-γ in developing Th2 cells 16.

TCF-1 is expressed in several isoforms that derive from different promoter usage and alternative splicing. These can roughly be divided into two groups. Fully functional, long TCF-1 isoforms arise from the distal promoter, whereas the proximal promoter produces short TCF-1 isoforms that lack the N-terminal β-catenin-binding domain and function as persistent transcriptional repressors 58. The study of Yu et al. 17 demonstrated that only long, fully functional TCF-1 isoforms initiate *GATA3–1b* transcription, whereas over-expression of a mutated TCF-1 construct in which the sequence encoding the β-catenin-binding domain was deleted had the opposite effect. As this mutated TCF-1 construct resembles the naturally occurring short TCF-1 isoforms, it is highly likely that short TCF-1 isoforms are natural suppressors of *GATA3–1b* and thus act as inhibitors of TCF-1-driven Th2 differentiation.

We recently reported that IL-4 is a highly effective inhibitor of the short, suppressive TCF-1 isoforms 59. We showed that minimal amounts of IL-4 are sufficient to inhibit TCF-1 expression in a STAT6-dependent fashion. Because long and short TCF-1 isoforms might have different functions in the regulation of *GATA3–1b* transcription, differential regulation of TCF-1 isoforms is of special relevance to Th2 polarization. Given that IL-4-mediated suppression of TCF-1 specifically targets the short TCF-1 isoforms and requires a minimal concentration of IL-4, the following model of an IL-4/STAT6-dependent fine-tuning mechanism operating at the initial phase of TCF-1-driven Th2 development can be proposed.

Upon TCR ligation, β-catenin translocates into the nucleus to activate, in association with long TCF-1 isoforms, expression of the early *GATA3* transcript *GATA3–1b*. However, naïve CD4+ T cells express not only the long TCF-1 isoforms that drive the expression of *GATA3* but also the short isoforms. As noted above, these short TCF-1 isoforms are suppressors of *GATA3*, and their effect is to limit GATA3 expression and, as consequence, IL-4 production as well. At this stage of Th2 development, IL-4 is produced in small amounts which are probably too low to activate STAT6-dependent *GATA3* transcription. However, we propose that IL-4 in low concentration is still able to effectively suppress the short TCF-1 isoforms. Thus, the *GATA3* locus is relieved of its repressor, allowing for higher transcription rates. Along with GATA3 expression, IL-4 production in developing Th2 cells is enhanced and the classical IL-4/STAT6-dependent Th2 polarization pathway enables manifestation of the Th2 phenotype 59.

## Conclusions

Differentiation of naïve CD4+ T cells into Th2 cells is a complex process involving IL-4/STAT6-dependent and -independent signals. In the context of Th2 differentiation, STAT6 was initially regarded as a transcriptional activator. However, it is now clear that STAT6 can also act as a repressor. STAT6-dependent suppression of naturally occurring inhibitors, as shown for repressive TCF-1 isoforms, clearly demonstrates that STAT6-dependent and -independent mechanisms are closely linked to each other. This further suggests that, though other pathways have been identified which also contribute to the development and function of Th2 cells, IL-4 and STAT6 are central players in this process.

## Abbreviations

DLL: delta-like ligand
GATA3: GATA-binding protein 3
LEF-1: lymphoid enhancer-binding factor 1
mTOR: mammalian target of rapamycin
TCF-1: T-cell factor 1
